# Epigenetic regulation of oligodendrocyte myelination in developmental disorders and neurodegenerative diseases

**DOI:** 10.12688/f1000research.20904.1

**Published:** 2020-02-11

**Authors:** Kalen Berry, Jiajia Wang, Q. Richard Lu

**Affiliations:** 1 Department of Pediatrics, Brain Tumor Center, Division of Experimental Hematology and Cancer Biology, Cincinnati Children’s Hospital Medical Center, Cincinnati, OH, 45229, USA

**Keywords:** epigenetics, developmental disorders, neurodegenerative disease, oligodendrocyte, myelination, myelin repair, multiple sclerosis, chromatin remodelers, histone-modifying enzymes, DNA methylation, RNA modification

## Abstract

Oligodendrocytes are the critical cell types giving rise to the myelin nerve sheath enabling efficient nerve transmission in the central nervous system (CNS). Oligodendrocyte precursor cells differentiate into mature oligodendrocytes and are maintained throughout life. Deficits in the generation, proliferation, or differentiation of these cells or their maintenance have been linked to neurological disorders ranging from developmental disorders to neurodegenerative diseases and limit repair after CNS injury. Understanding the regulation of these processes is critical for achieving proper myelination during development, preventing disease, or recovering from injury. Many of the key factors underlying these processes are epigenetic regulators that enable the fine tuning or reprogramming of gene expression during development and regeneration in response to changes in the local microenvironment. These include chromatin remodelers, histone-modifying enzymes, covalent modifiers of DNA methylation, and RNA modification–mediated mechanisms. In this review, we will discuss the key components in each of these classes which are responsible for generating and maintaining oligodendrocyte myelination as well as potential targeted approaches to stimulate the regenerative program in developmental disorders and neurodegenerative diseases.

## Introduction

Oligodendrocytes are the specialized glial cells of the central nervous system (CNS) that produce the myelin sheaths surrounding axons and enabling salutatory conduction as well as providing metabolic support to axons ^1^. Defects in the myelination process have been associated with developmental disorders such as autism ^2– 5^ and coloboma, heart disease, atresia choanae, retarded growth and development, genital hypoplasia, and ear abnormalities (CHARGE) syndrome ^6, 7^ as well as neurodegenerative diseases such as the demyelinating disease multiple sclerosis (MS) and various leukodystrophies ^8^. The late-onset neurodegenerative diseases may also stem from subtle dysregulation of early developmental processes. In addition, dysregulation of the processes controlling proliferation and differentiation in the oligodendrocyte lineage has been linked to the development of various brain cancers ^9^. Understanding developmental myelination and remyelination processes will have impacts for the development of treatments to improve functional recovery after injury or disease ^10– 13^.

**Figure 1.**
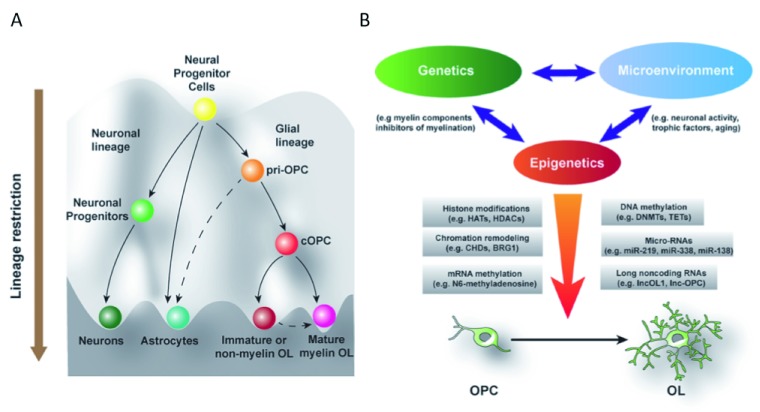
Differentiation of progenitor cells is a highly choreographed process. ( **A**) A diagram depicts an epigenetic landscape of cellular fate decision-making during oligodendrocyte development from neural progenitor cells. Beginning with neural progenitors, cell differentiation occurs along multiple potential pathways with cells taking on neuronal, astrocyte, or oligodendrocyte lineages. This differentiation from a common progenitor population involves the fine tuning of gene expression and turning on and off of lineage-specific genes and their epigenetic regulators. ( **B**) Many modulators of gene expression are through epigenetic mechanisms, which alter gene expression on the basis of local environmental factors. These mediators include covalent modifications to DNA or histones, RNA-mediated regulation of gene expression, or the enzymes responsible for mediating the effects of these modifications. BRG1, Brahma-related 1; CHD, chromodomain helicase DNA-binding; cOPC, committed oligodendrocyte progenitor cell; DNMT, DNA methyltransferase; HAT, histone acetyltransferase; HDAC, histone deacetylase; OL, oligodendrocyte; pri-OPC, primitive oligodendrocyte progenitor cell; TET, ten-eleven translocation.

The oligodendrocyte lineage originates from multi-potent neural progenitor cells (NPCs). Early NPC divisions result predominantly in neurons before switching to a primarily glial progeny later in development ^14– 16^. First, NPCs become primitive oligodendrocyte progenitor cells (pri-OPCs or pre-OPCs) expressing Olig1/2, then committed OPCs (PDGFRα ^+^/NG2 ^*+*^), which persist in the CNS throughout life ^17^. OPCs can further proliferate and differentiate into mature myelinating oligodendrocytes ^18– 21^. The transition into each of these stages requires the coordination of intrinsic and extra-cellular cues where transcriptional regulatory events are closely interconnected and function together to safeguard the oligodendrocyte identity and prevent alternative cell fates such as astrocytes or neurons ( Figure 1A).

The oligodendrocyte lineage is highly responsive to environmental cues. For example, activity or experience can promote myelination of axons by newly formed oligodendrocytes and even induce the proliferation of OPCs ^22– 29^. Additionally, there exist critical periods during oligodendrocyte development and myelination ^30, 31^ when oligodendrocytes are highly receptive and adaptive to environmental cues such as neuronal activity ^32^. The plasticity of myelinating oligodendrocytes and adaptive myelination are important for normal neural circuit function and cognition ^33^. Epigenetic regulation is likely the process through which the effects of these kinds of stimuli are carried out. At present, how epigenetic mechanisms mediate the environmental cues for oligodendrocyte myelination and remyelination remains poorly defined.

In recent years, the importance of epigenetic mechanisms and their non-genetic regulation of gene expression and cell states has been increasingly recognized ^18, 20, 34, 35^ ( Table 1). Epigenetic regulation of gene expression occurs through a variety of mechanisms, including covalent modifications of chromatin to regulate stearic access to DNA, ATP-dependent nucleosome remodeling, DNA methylation, non-coding RNAs, and RNA modifications ^21, 78^. All of these processes can modulate large-scale genetic programs to alter and maintain cell states during oligodendrocyte progenitor proliferation and maturation ( Figure 1B). Epigenetic modifications are often reversible and provide the necessary plasticity for progenitor cells to respond to environmental cues. Such pathways are amenable to pharmacological intervention and could be targeted to promote myelin growth or repair.

**Table 1.** Epigenetic pathways in oligodendrocyte development and myelination.

Epigenetic regulators	Component	Description	Function in oligodendrocytes
**ATP-dependent** **chromatin remodelers**	BRG1 (also known as Smarca4)	A key helicase subunit of the SWI/SNF Family	Stage-dependent promotion of OPC differentiation but not required for OPC survival ^36, 37^.
	CHD7	Member of the chromo helicase domain family	CHD7 is required for oligodendrocyte differentiation and remyelination in the spinal cord ^6, 38, 39^.
	CHD8	Member of the chromo helicase domain family	CHD8 has been linked to autism disorder with white matter defects. CHD8 knockout in the oligodendrocyte lineage leads to myelination defects ^40, 41^.
**Histone acetylation** **modifiers**	EP300 (also known as p300)	Histone acetyltransferase	Associated with Rubinstein–Taybi syndrome ^42, 43^ and regulates oligodendrocyte differentiation ^44^.
	EP400 (E1A Binding Protein P400)	Key subunit of TIP60 histone acetyltransferase complex	Deletion in CNP ^+^ oligodendrocytes leads to defects in terminal differentiation and hypomyelination ^45^.
	HDAC1	Class I histone deacetylase (HDAC)	Regulates oligodendrocyte differentiation via co-repressor complexes ^46, 47, 48^ and has non– histone-dependent effects in oligodendrocyte differentiation ^49^.
	HDAC2	Class I HDAC	Functionally redundant regulation of oligodendrocyte differentiation with HDAC1 ^46^.
	HDAC3	Class I HDAC Complexes with co- repressors NCOR/SMRT	Regulates the fate choice of primitive OPCs between astrocytic and oligodendrocytic fates and myelination ^44^.
	SIRT1	Class III NAD ^+^ HDAC	Stage-dependent effects on OPC proliferation. Increased OPC differentiation when knocked out in OPCs ^50^.
	SIRT2	Class III NAD _+_ HDAC	Highly expressed in mature oligodendrocytes. Its level is positively correlated with oligodendrocyte differentiation ^51^.
	HDAC6	Class II HDAC	Regulates oligodendrocyte differentiation via is acetylation of tubulin in the cytoskeleton ^52^.
	HDAC10	Class II HDAC	No clear role, likely due to functional redundancy with other HDACs in its regulation of OLIG1 nuclear localization ^49^.
	HDAC11	Class IV HDAC	Regulates oligodendrocyte differentiation possibly via modulating regulatory elements of myelin- related genes ^53, 54, 55^.
**Histone methyl-** **transferases**	COMPASS-like complex	Major subunits include SETD1A, MLL1, and MLL2 (KMT2A)	MLL2 works with CHD8 to deposit H3K4me3 at active promotors of oligodendrocyte lineage genes ^41^.
	PRC2 complexes	Major subunits include EZH2, EED, and SUZ12	Responsible for H3K27me3 deposition. Promotes oligodendrogenesis and OPC differentiation ^56, 57^.
	PRMT1	Catalyzes histone arginine methylation	Required for proper OPC differentiation resulting in hypomyelination defects ^58^.
	PRMT5	Catalyzes histone arginine methylation	Required for proper OPC differentiation resulting in hypomyelination defects ^59– 61^.
**DNA methyl-** **transferases and** **demethylases**	DNMT1	DNA methyltransferase	Knockout early development impairs OPC differentiation and results in hypomyelination ^62^. Has no effect on myelin repair ^55^.
	DNMT3a	DNA methyltransferase	Plays a role in myelin repair after injury but not early development of the oligodendrocyte lineage ^55^.
	TET1–3 (ten-eleven translocation)	DNA demethylases that catalyze the conversion of 5mC to 5hmC	Differentially regulated at different stages during OL development. Tet1 is required for OL differentiation *in vitro* ^63^.
**microRNAs**	Dicer	Enzyme responsible for processing microRNAs into mature form	Required for OPC differentiation, myelination, and myelin maintenance ^64– 66^.
	*miR-219*		miR-219 is necessary and sufficient to induce differentiation ^65, 66^. Also required for remyelination after lysophosphatidylcholine (LPC)-induced demyelination ^67^.
	*miR-338*		miR-338 is dispensable for OPC differentiation or myelination *in vivo* but has synergistic with miR-219 ^67^.
	*miR-212*		Negatively regulates common oligodendrocyte and myelin-related genes by miR-212 ^68^.
	*miR-125a-3p*		Upregulated in cerebrospinal fluid from multiple sclerosis patients with active demyelinating lesions. Negatively regulates oligodendrocyte differentiation ^69^.
**Long non-coding** **RNAs**	*lncOL1*		LncOL1 positively regulates OPC differentiation while having no effect on OPC formation. Affects timing of myelinogenesis but not the maintenance of myelin ^56^.
	*Lnc-OPC*		Knockdown of lnc-OPC in NPCs limited their differentiation into OPCs without affecting NPC proliferation ^70^.
	*Pcdh17it*		A marker of the immature premyelinating oligodendrocyte population ^71^.
	*SOX8OT*		Regulates oligodendrocyte differentiation through targeting SOX8 ^72, 73^.
	*Neat1*		Knockout reduces the number of oligodendrocytes in the frontal cortex ^74^.
	*Lnc158*		Correlates with oligodendrocyte differentiation- associated gene expression ^75^.
*N* **^6^-methyl-adenosine** **(m ^6^A) modifiers**	METTL14	m6A RNA writer	Required for OPC differentiation and proper myelination ^76^.
	PRRC2A	An m6A RNA binding protein	Highly expressed in OPCs and white matter tracks. Required for normal OPC proliferation and differentiation ^77^.
	FTO	m6A RNA demethylase (alpha-ketoglutarate– dependent dioxygenase)	Knockout mimics the effects of PRRC2A overexpression increasing Olig2 expression ^77^.

CHD, chromodomain helicase DNA-binding; HDAC, histone deacetylase; OL, oligodendrocyte cell line; OPC, oligodendrocyte progenitor cell; NPC, neural progenitor cell.

## ATP-dependent chromatin remodelers in oligodendrocyte lineage progression and regeneration

**Figure 2.**
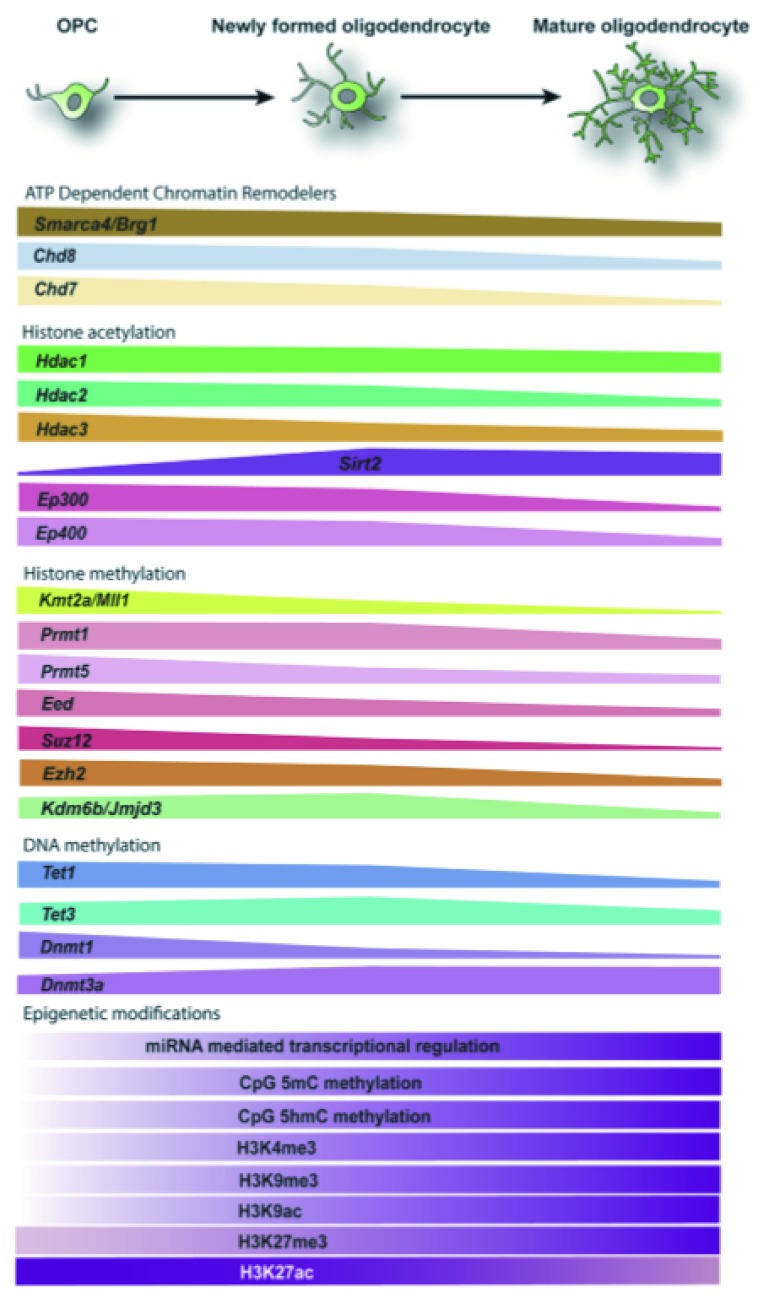
Global expression levels of key epigenetic regulators during oligodendrocyte differentiation from progenitor cells. Epigenetic modifiers, including ATP-dependent chromatin remodelers, histone acetyltransferases and deacetylases, histone methyltransferases, and demethylases, are critical components of the differentiation process, according to the data from a bulk RNA sequencing dataset ^92^. The change of epigenetic modifiers across oligodendrocyte differentiation is depicted. The global changes of expression levels in the epigenetic modifications themselves are based on the studies ^41, 44, 62, 63, 66, 93^. OPC, oligodendrocyte progenitor cell.

ATP-dependent chromatin remodeling uses ATP to remodel the nucleosome, opening up areas for enhancing transcription, and is critical for neural cell growth and differentiation ^79, 80^. Early work in cell cultures showed that OPCs differentiating into mature oligodendrocytes underwent substantial chromatin reorganization within the nucleus ^81^. The chromatin remodelers consist of several multi-subunit complexes which fall into four major families: the SWI/SNF family with the major ATPase subunits Brahma-related 1 (BRG1, also known as Smarca4) and Brahma (BRM, also known as SMARCA2), the INO80 family which includes the ATPases INO80 and SRCAP, the ISWI family with ATPase subunits SNF2L and SNF2H, and the chromodomain helicase DNA-binding (CHD) family consisting of CHD1–9 ^80, 82, 83^. Of these, the SWI/SNF family and the CHD family members are dynamically regulated over the course of OPC specification and differentiation and have been implicated in oligodendrocyte development and myelination ( Figure 2).

### SWI/SNF family members

The SWI/SNF family of ATPase dependent chromatin remodelers have been shown to play critical roles in the development of the oligodendrocyte lineage. Deletion of *Brg1/Smarca4*, the core helicase component of the SWI/SNF family, in NPCs inhibits oligodendrocyte and astrocyte lineage development while increasing neuronal differentiation in the ventricular zone of the developing brain ^80, 84^. A lineage-specific transcription factor, OLIG2, can recruit the BRG1/SWI/SNF complex to the promoters and enhancers of oligodendrocyte lineage genes such as *Sox10* to activate their transcription. BRG1 is also necessary for OPC differentiation. BRG1 expression increases after induction of rat OPC differentiation with T3 thyroid hormone ^36^. These increasing levels are critical for OPC differentiation as conditional knockout in *Olig1*-expressing oligodendrocyte progenitors and PDGFRa-expressing OPCs *in vivo* leads to oligodendrocyte differentiation defects and profound dysmyelination defects ^36^ (JW and QL, unpublished). Of note, the loss of *Brg1* does not affect OPC survival in culture or *in vivo* ^36^. However, *Brg1* knockout in later OPCs, such as NG2 ^+^ or CNP ^+^, committed or post-mitotic OPCs, respectively, had progressively less severe effects on differentiation ^37^, suggesting that BRG1 effects are stage-dependent. This stage-dependent severity suggests that BRG1 activates early pro-differentiation factors, such as SOX10, that can continue to mediate downstream genetic programs in oligodendrocyte lineage progression despite the upstream loss of BRG1 ^36, 37^. In addition, other chromatin remodelers such as CHD8 or CHD7 (discussed below) potentially compensate for the loss of BRG1 at later stages.

### CHD family members

CHD7 is highly enriched in the oligodendrocyte lineage, especially in differentiating oligodendrocytes. *CHD7* mutations result in a series of birth defects called CHARGE syndrome, which exhibits impaired white matter development and myelination in addition to other congenital developmental abnormalities ^85, 86^. CHD7, like BRG1 above, does not affect OPC formation but instead causes defects in OPC differentiation ^6, 38, 39^. In fact, *Chd7* is a direct target of the OLIG2/BRG1 complex and its expression is greatly increased by the binding of this complex at its promoter ^6^. CHD7 can complex with SOX10 to activate downstream regulators of oligodendrocyte differentiation. CHD7 activates the expression of OPC pro-differentiation regulators, including SOX10 and NKX2-2 ^39^, as well as other oligodendrocyte-expressing transcription factors such as Osterix/Sp7 and Creb3l2 ^6, 39^. Intriguingly, deletion of Chd7 in PDGFRa ^+^ OPCs appears to impair OPC survival via p53 upregulation ^39^. CHD7 binds to the *p53* promotor in OPCs and limits p53 expression to maintain the survival of OPCs ^39^.

CHD7 is also required for remyelination after lysolecithin-induced demyelination ^6^ or spinal cord laminectomy, wherein it interacts with SOX2 to drive OPC differentiation ^38^. *Chd7* deletion impairs OPC proliferation after spinal cord injury ^38^ but not in the developing brain ^6, 39^, suggesting a context-dependent CHD7 regulation of OPC proliferation. However, CHD7 appears to be dispensable for the maturation of oligodendrocytes, possibly due to compensation by other CHD members such as CHD8 ^39^, which has been shown to work together with CHD7 to regulate OPC survival and maturation ^39^.

Another CHD family member, CHD8, is also critical for proper oligodendrocyte development. CHD8 has been linked to a subset of autism disorders, which exhibit a defect in white matter tracts and myelination ^40, 41, 87– 89^. *Chd8* knockout in *Olig1* ^+^ progenitors causes defects in CNS myelination, particularly in the spinal cord because of severe reductions in PDGFRα-expressing OPCs in this region ^41^, suggesting a region-specific role of CHD8 in OPC survival and differentiation. Deletion of *Chd8* at the post-natal stages with an inducible PDGFRα-CreER driver also blocks OPC differentiation. The defects in oligodendrocyte differentiation are due to the cell-specific loss of *Chd8* in the oligodendrocyte lineage as there are no defects seen after *Chd8* knockout in post-mitotic neurons ^41^. This suggests that the myelination defects seen in *CHD8* mutant patients are cell-autonomous defects due to the loss of *CHD8*. Remyelination after lysophosphatidylcholine (LPC)-induced demyelinating lesions in the spinal cord is also dependent on CHD8 expression ^41^. CHD8 dysregulation may be an important factor for white matter pathogenesis and remyelination failure given the critical role of CHD8 for OPC replenishment and remyelination in demyelinating lesions.

CHD7 and CHD8 have similar structures and can bind many of the same targets. However, they target different gene regions during oligodendrocyte differentiation. CHD7 predominantly binds to promotor regions in OPCs but switches to enhancer regions in oligodendrocytes ^39^. CHD8, in contrast, binds predominantly to promotor elements near transcription start sites marked by an activating histone mark H3K4me3 in OPCs and oligodendrocytes where it recruits an H3K4 activating histone methyltransferase MLL2 (mixed lineage leukemia 2) complex to drive expression of oligodendrocyte lineage genes ^41^. MLL2–4 and other family members can form a macromolecular complex called COMPASS (complex of proteins associated with Set1) to methylate H3K4 and regulate gene transcription ^90^. Strikingly, blocking lysine demethylase KDM5, an enzyme that erases methylation on H3K4 ^91^, with a pan-KDM5 inhibitor CPI-455 rescues the differentiation defects in *Chd8* mutant OPCs ^41^, suggesting that targeting this eraser to enhance H3K4me3 levels might facilitate the restoration of myelination defects caused by CHD8 defects.

The chromatin remodelers may all work together to regulate oligodendrocyte development but each has its own preferences for regulatory elements and mechanisms to control expression of specific sets of targeted genes. Of these, CHD8 appears to turn on the earliest, eventually promoting BRG1 expression which in turn induces CHD7 expression ^6^. This successive signaling cascade enables the progression from OPCs to mature myelinating oligodendrocytes and likely forms convergent points upon which other checkpoints and regulatory mechanisms act to facilitate this development. Nonetheless, these chromatin remodelers could operate simultaneously in a non-linear fashion to promote oligodendrocyte lineage progression.

## Histone acetylation control of cell fates and differentiation in the oligodendrocyte lineage

Histone acetylation, in particular, has been strongly implicated in the regulation of oligodendrocyte development. The addition and elimination of acetyl groups are balanced through the competing work of histone acetyltransferases (HATs) ^94^ and histone deacetylases (HDACs).

### HATs

The activity of HATs is responsible for the acetylation of histones, leading to relaxed chromatin coiling and increased gene expression. The acetylation of histone H3 on lysine 27 (H3K27ac) is often deposited at active regulatory elements such as enhancers and promoters and is positively correlated with the activation of gene transcription ^95^. H3K27 acetylation is catalyzed by multiple HATs, including p300 (also known as EP300), CREB-binding protein (CBP), TIP60, and PCAF ^96, 97^. Histone acetylation status can be further recognized by bromo-, PHD-, Tudor-, or WD40-domain-containing transcription activating regulators, which further modulate target gene expression ^95, 98– 100^.

In line with a critical role for histone acetylation in regulating the oligodendrocyte lineage, the loss of multiple HATs can lead to defects in the myelination process. Deletion of EP400, a key subunit of the TIP60 HAT complex, in *Cnp*-expressing oligodendroglial cells results in a defect in oligodendrocyte terminal differentiation, leading to profound hypomyelination ^45^. A genetic disorder, Rubinstein–Taybi syndrome, is associated with mutations in p300 (also known as EP300), which is characterized in part by hypoplasia of the corpus collosum and congenital hypomyelination ^42, 43^. The role of p300 in controlling oligodendrocyte development is still being explored, but p300 has been shown to interact with HDAC3 (discussed in more detail below) partly facilitating the role of HDAC3 in promoting oligodendrocyte as opposed to astrocytic cell lineages during early differentiation from NPCs via its promotion of *Olig2* expression ^101^. Also, inhibition of p300 activity itself can lead to pronounced defects in OPC differentiation (JW and QL, unpublished).

### HDACs

Histone acetylation status can be reversed by HDACs. Mammals possess four classes of HDACs. Class I contains HDACs 1–3 and 8, class II contains HDACs 4–7 and 9 and 10, class III are NAD-dependent HDACs (also known as sirtuins, encompassing SIRT1–7), and finally class IV contains one HDAC, HDAC11 ^102, 103^. Pharmacological studies using HDAC inhibitors have indicated the importance of HDACs in oligodendrocyte fate specification and differentiation. Treating rat NPCs with valproic acid inhibited oligodendrogenesis and astrogenesis while promoting neurogenesis likely through NeuroD1 upregulation following the inhibition of HDAC activity ^104^. Blocking HDAC activity with pan inhibitors also disrupts the differentiation of OPCs into mature oligodendrocytes ^105^. The timing of this treatment appears to be critical. Treating cells with pan-HDAC inhibitors after the differentiation process has been shown to have minimal effect on oligodendrocyte differentiation ^106^. These studies indicate that HDACs play various roles at different stages during OPC differentiation and subsequent myelination. However, classic pan-HDAC inhibitors are non-specific and target HDACs across multiple classes. Genetic manipulation can specifically target individual HDACs to define their specific functions during oligodendrocyte lineage progression.

### Class I HDACs

The expression levels and functions of class I HDACs are important for oligodendrocyte fate specification and differentiation ( Figure 2). HDAC1 and HDAC2, when knocked out individually in the oligodendrocyte lineage, have no obvious effects on OPC formation, proliferation, or differentiation ^46^. However, the double-knockout animals die shortly after birth, and analysis revealed a severe defect in OPC proliferation or differentiation in these animals, suggesting that HDAC1 and HDAC2 can functionally compensate for the loss of the other in oligodendrocyte lineage determination ^46^. Another HDAC class I family member, HDAC3, has been implicated in the control of oligodendrocyte lineage specification ^44^ but differs from those effects observed in HDAC1. HDAC3 deletion at the same stages as HDAC1 and 2 above results in a switch from oligodendrocyte to astrocytic fates, suggesting that HDAC3 regulates the fate choice of primitive OPCs between astrocytic and oligodendrocytic cell lineages ^44^.

HDACs have been shown to exhibit non–histone dependent functions during oligodendrocyte development. HDAC1/2 co-repressor complexes can compete with β-catenin for binding to TCF7L2 (TCF4), a member of the TCF transcription factor family, resulting in the disinhibition of TCF7L2, which then is free to promote OPC differentiation ^46, 47, 48^. This is an example of how non-enzymatic activity of HDACs through protein–protein interaction in addition to the deacetylase activity can function to regulate genetic expression. HDAC1 can also be recruited by YY1 transcription factor to the promotor of oligodendrocyte differentiation inhibitory genes such as *Id4* to reduce their expression ^107, 108^. In addition, HDAC1 and HDAC3 can deacetylate the OLIG1 transcription factor, increasing its likelihood of nuclear translocation and ultimate promotion of OPC differentiation ^49^.

HDAC activity can be modulated through co-regulators or covalent modifications ^109^. For example, casein kinase 2 (CK2) phosphorylates HDAC3 to activate its activity while phosphatase 4 dephosphorylates it ^110^. Of interest, *in vitro* experiments revealed that the CK2 kinase, which activates HDAC3, also elevates expression of OLIG2, a critical transcription factor for initiating oligodendroglial cell fate ^111^. HDAC3 also forms protein complexes with co-repressor complexes such as NCOR and SMRT to regulate its activity ^112^. NCOR has been shown to negatively regulate astrogenesis through inhibiting JAK-STAT signaling, activation of which leads to astrocyte differentiation ^113^. In addition, HDAC3/NCOR can deacetylate and inactivate astrocyte-promoting factor STAT3 and therefore promote oligodendrogenesis while inhibiting the astroglial fate ^44^. HDAC3 also forms complexes with the HAT p300, to regulate OLIG2 expression levels during OPC specification ^44^. This interaction likely indicates that the coordinated activity of two opposing factors is required for oligodendrocyte and astrocytic lineage fate decisions.

HDAC3 functions not only as a transcriptional co-repressor, as one may assume from its histone deacetylation activity, but also as a transcriptional co-activator as in its role in the activation of retinoic acid response elements ^114, 115^. It is worth noting that HDAC3 deacetylase activity may not be vital for oligodendrocyte development. HDAC3 requires NCOR and SMRT to promote its deacetylation activity. Deleting the deacetylase-activating domains (DADs) in NCOR and SMRT abrogates the deacetylase activity of HDAC3. However, the DAD deletion mice survive to adulthood and exhibit normal myelination whereas the ablation of HDAC3 is embryonic lethal ^116^. Although the function of HDAC3 catalytic site mutants remains to be determined, the current data suggest that HDAC3 may serve as a scaffold for multi-component transcriptional regulatory complexes vital for oligodendrocyte myelination.

### Other HDAC classes

Among class II HDACs, HDAC6 has been shown in rat oligodendrocyte cultures to acetylate the microtubule-associated protein tau and α-tubulin, both of which are required for normal oligodendrocyte development ^52^. HDAC10, along with HDAC1 and HDAC3, has also been shown to regulate the nuclear localization of OLIG1 for oligodendrocyte maturation ^49^. It is worth noting that the enzymatic activity of class II HDACs is dependent on the HDAC3/SMRT/N-CoR complex ^117^.

The class III HDACs SIRT1 and SIRT2 have been shown to regulate early oligodendrocyte lineage determination ^50, 51, 118^. SIRT2, in particular, is highly expressed in mature oligodendrocytes ^119^ and regulates the differentiation of oligodendrocytes as blocking its activity or overexpressing it prevents or promotes differentiation of CG4 oligodendroglial cells, respectively ^120– 122^. This class of HDACs also relies on NAD as a co-factor for deacetylase activity ^123^. The loss of NAMPT, the rate-limiting enzyme for NAD biosynthesis in mammals, leads to defective oligodendrocyte development ^118, 124^. Like those of many other epigenetic factors, the effects that SIRT1 and SIRT2 have on development are stage-dependent. For example, *Sirt1* knockout in NPCs increases OPC proliferation ^50^ while *Sirt1* knockout in PDGFRa ^+^ OPCs promotes cell cycle exit and OPC differentiation ^125^. Notably, SIRT2 is depleted in myelin sheathes of PLP-deficient oligodendrocytes, a model for spastic paraplegia, suggesting that SIRT2 might have a role in myelin sheath maintenance and provide trophic support of axons ^126^.

Finally, the class IV HDAC, HDAC11 has been shown to regulate H3K9 and H3K14 acetylation and expression levels of *Mbp* and *Plp* genes ^53, 54^. HDAC11 overexpression enhances the maturation of an oligodendrocyte cell line (OL-1) *in vitro* ^53, 54^, suggesting a potential role of HDAC11 in regulating myelin gene expression. At present, how the function of each HAT and HDAC is controlled, individually and coordinately, on a system-wide level to regulate the complex processes of oligodendrocyte development and myelination remains to be defined. This is of particular importance given the reiterative involvement of many HAT and HDAC enzymes in the gene regulatory network during CNS development and regeneration.

## Histone methylation regulates oligodendrocyte differentiation

Histone methylation can be linked to either gene activation or gene repression. The activating histone mark H3K4 trimethylation (H3K4me3) is deposited mainly at promoter elements and associated with gene transcription ^127^. The COMPASS-like complex, consisting of SETD1A and MLL1/2, is a major enzyme responsible for H3K4me3 deposition ^128, 129^, although its function in oligodendrocyte development has not been fully defined.

During differentiation from a more plastic state to a more differentiated state, the level of repressive histone marks, for example, H3K27me3 and H3K9me3 increases across many different cell types ^130, 131^, including oligodendrocyte lineage cells ^56, 93^. The histone methyltransferases mediating the deposition of these marks are critical in the differentiation of oligodendrocytes. Inhibition of H3K9 histone methyltransferases in cell culture via pharmacological inhibitors or shRNAs suggested a role of H3K9 deposition in the progression of the OL lineage and the suppression of neuronal gene programs ^93^. However, the *in vivo* role of these H3K9 histone methyltransferases in oligodendrocyte development remains to be defined.

The importance of H3K27 trimethylation (H3K27me3) in oligodendrocyte development is more defined. Polycomb repressive complex 2 (PRC2), consisting of EZH2, EED, and SUZ12, is the sole enzyme responsible for H3K27me3 in mammals ^132– 134^. Expression of PRC2 complex components exhibits a spatiotemporal-specific pattern ^135– 137^, suggesting that individual PRC2 subunits may play distinct functions during oligodendrocyte development and myelination. EZH2, the core catalytic subunit of PRC2 mediating its methyltransferase activity, promotes oligodendrogenesis from neural stem cells as opposed to astrocyte formation in a dose-dependent manner ^57^. In addition, the loss of *Ezh2* at later stages in *Olig1*-expressing progenitors prevents OPC differentiation, decreasing the number of mature oligodendrocytes *in vivo* ^56^. These observations suggest that elevation of H3K27me3 levels is required for oligodendrocyte differentiation. Of note, mutations in the histone such as H3.3K27M precludes PRC2-mediated H3K27me3 ^138, 139^. This mutation limits the capacity for OPC differentiation and is a major factor contributing to the development of malignant diffuse intrinsic pontine glioma (DIPG) ^138, 140, 141^. OPCs or pri-OPCs have been implicated as the tumor cells of origin for H3K27M midline gliomas ^142, 143^, highlighting the critical nature of this epigenetic mechanism in regulating the development of the oligodendrocyte lineage.

Another histone methyltransferase family that catalyzes arginine instead of lysine methylation, PRMTs ^144^, is also implicated in OPC differentiation. PRMT1 ^58^ and PRMT5 ^59– 61^ have both been shown to be required for proper differentiation of OPCs into mature oligodendrocytes, and loss-of-function mutants develop hypomyelination phenotypes. The function of other PRMT family members in oligodendrocyte myelination remains to be further defined. Overall, these studies demonstrate that the balance of histone methyltransferases and histone demethylases is likely critically important for the regulation of oligodendrocyte development and remyelination.

## DNA methylation and demethylation in oligodendrocyte development

DNA methylation is an epigenetic regulatory mechanism where cytosines, specifically those preceding guanine in so-called CpG islands, are methylated. CpG islands are preferentially found in the 5′ promotor region of genes and their methylation state can inhibit or promote the expression of the relevant gene ^145^. The methylation status of these sites is regulated by the coordinated activity of DNA methyltransferases (DNMTs), which add methyl groups to convert cytosine into 5-methylcytosine, and ten-eleven translocation (TET) proteins or DNA demethylases, which catalyze the conversion of 5-methylcytosine to 5-hydroxymethylcytosine (5hmC), beginning the process of converting 5-methylcytosine back to cytosine ^146^. The expression of individual DNMTs and TETs varies across the OL lineage, suggesting a potential stage-specific role of DNMTs and TETs for oligodendrocyte development, myelination, and remyelination. In line with these observations, there was also a significant increase in DNA methylation during OL maturation ^62^.

DMNT1 is downregulated during oligodendrocyte differentiation where other DMNT family members had no change ^62^. Deletion of *Dmnt1* early in the oligodendrocyte lineage had a profound effect on oligodendrocyte differentiation, resulting in hypomyelination *in vivo* ^62^. This effect was not due to the upregulation of normally methylated genes alone; defects in alternative splicing mediated by DNA methylation were also attributed to the failure in myelination ^62^. In contrast to the *Dmnt1* knockout, *Dmnt3a* knockout in NPCs had no effect ^62^. However, after lysolecithin-induced demyelination, tamoxifen-inducible *Dnmt3a* deletion in mature oligodendrocytes using a PLP-CreERT2 driver line impaired remyelination whereas the inducible *Dnmt1* knockout had no effect ^147^. Taken together, these results suggest that in some cases remyelination in adulthood does not fully recapitulate the developmental program.

TET1, TET2, and TET3 have all been implicated in the differentiation of oligodendrocytes *in vitro* ^63^. However, they each have different structures and their expression and subcellular localizations differ ^63^, suggesting that they may play different roles in regulating oligodendrocyte differentiation. TET1 is downregulated in mature oligodendrocytes, TET2 translocates from the cytoplasm to the nucleus during OPC differentiation, and TET3 is seen only in the nucleus of maturing oligodendrocytes ^63^. Of these, TET1 appears to show the strongest effect in regulating the oligodendrocyte lineage where the knockout impairs oligodendrocyte development and remyelination after lysolecithin-induced demyelination ^148, 149^.

## Non-coding RNAs in oligodendrocyte development and myelination

Non-coding RNAs such as microRNAs (miRNAs) or long-non-coding RNAs (lncRNAs) play regulatory roles in oligodendrocyte development, myelination, and remyelination. miRNAs are short RNA sequences that bind to homologous sequences on mRNA transcripts to inhibit translation into proteins. These miRNAs are processed into their mature active form by the enzyme Dicer. Conditional deletions of Dicer in OPCs and mature oligodendrocytes have all resulted in defects in myelination. Despite the myelin defects, the population of proliferating OPCs is increased in these animals, indicating a critical role for miRNAs in balancing OPC proliferation and differentiation ^64– 66^. Post-natal Dicer1 ablation in mature oligodendrocytes results in demyelination and oxidative damage, leading to neuronal degeneration and inflammatory astrogliosis and microgliosis in the brain ^64^, suggesting a critical role of Dicer and thus miRNAs in myelin lipid maintenance and redox homeostasis.

### miRNAs

Comparisons between OPCs and immature and mature oligodendrocytes revealed a set of miRNAs enriched during oligodendrocyte differentiation, including miR-219, miR-138, and miR-338 ^64– 66, 150^. miR-219 is necessary and sufficient to induce differentiation and can even partially rescue the Dicer knockout phenotype ^65, 66^. Knockout of miR-219–encoding genes ( *miR-219-1* and *miR-219-2*) led to reduced myelination throughout the CNS ^67^. In contrast to miR-219, miR-338 is dispensable for OPC differentiation or myelination *in vivo*. However, there was a synergistic effect on the myelination defects in miR-219 and miR-338 double-conditional knockout mice. miR-219 is also required for remyelination after LPC-induced demyelination. Overexpression of miR-219 in OPCs increased oligodendrocyte differentiation and could even promote repair when overexpressed genetically or administered with intrathecal injections of miR-219 mimics ^67^. miR-219 likely functions by repressing inhibitors of OPC differentiation, including Lingo1 and Etv5 ^67^. miR-219 has been suggested in zebrafish to regulate oligodendrocyte lineage specification from NPCs ^151^. However, there were no defects of OPC specification in the *miR-219-1/2* double-null animals ^67^, suggesting a species-specific effect. Other miRNAs have been associated with oligodendrocyte development (reviewed in more detail in 152), but of these miR-219 exhibits the strongest effects.

A set of miRNAs have been identified to negatively regulate oligodendrocyte differentiation. One of these, miR-212 was found to be downregulated in oligodendrocytes after spinal cord injuries in rats, where it appears to repress expression of differentiation-associated genes ^68^. Another such miRNA, miR-125a-3p, was enriched in cerebrospinal fluid from MS patients with active demyelinating lesions. miR-125a-3p overexpression impaired oligodendrocyte differentiation whereas knocking it down promoted differentiation ^69^. Similarly, overexpression of miR-27a inhibits oligodendrocyte differentiation and myelination by activating the Wnt/beta-catenin signaling pathway ^153^. Such negative regulatory miRNAs are potential targets for enhancing remyelination.

### LncRNAs

LncRNAs are long RNA sequences (more than 200 nucleotides) that are highly conserved across species but have no coding potential ^154^. LncRNAs have been implicated in the regulation of both normal development ^155, 156^ and diseases ^157, 158^. LncRNAs can be very specifically expressed in the oligodendrocyte lineage; for instance, *lncOL1* and *Pcdh17it* were recently identified as specific markers for oligodendrocytes and immature premyelinating oligodendrocytes, respectively ^56, 71^. Gene-chip microarrays were initially used to identify lncRNAs such as SOX8OT (SOX8 opposite transcript) in cultured OPCs. SOX8OT might have a role in regulating oligodendrocyte differentiation through its regulation of SOX8 ^72, 73^.

Combining RNA sequencing and chromatin mapping across oligodendrocyte lineage stages revealed several lncRNAs that are actively transcribed and restricted to this lineage ^56^. Of these, *lncOL1* was identified as a top candidate on the basis of its abundance, regulation during oligodendrocyte differentiation, and preliminary screening for effects on myelin-associated gene expression. *lncOL1* overexpression led to precocious oligodendrocyte differentiation in mouse embryos, and *lncOL1* knockout led to defects in OPC differentiation while having no effect on OPC formation. Interestingly, these myelination defects were seen only during development but not in adulthood, suggesting a role of *lncOL1* in regulating the timing of myelinogenesis and not the maintenance of myelin. *lncOL1* mediates this effect in part by its interaction with SUZ12, a member of the PRC2 complex which mediates histone methylation through EZH2. *lncOL1* directs the PRC2 complex to silence the expression of OPC-associated genes via H3K27me3 deposition ^56^. In contrast to *lncOL1*, *lnc-OPC*, another lncRNA found in the oligodendrocyte lineage, is enriched in OPCs and regulated by OLIG2 ^70^. Knockdown of *lnc-OPC* in cultured NPCs limited their differentiation into OPCs without affecting NPC proliferation ^70^. In similar fashion, *lnc158* expression directly correlated with oligodendrocyte-associated protein expression and differentiation along the oligodendrocyte lineage ^75^. In addition, another lncRNA, *Neat1*, was downregulated in schizophrenia. *Neat1* knockout mice exhibited a reduction in the number of oligodendrocytes in the frontal cortex because of a failure in the retention of oligodendrocyte transcription factors in the nucleus ^74^. These studies indicate that lncRNAs regulate oligodendrocyte development and myelination via various processes such as controlling mRNA transcripts, nuclear localization of transcription factors, or interactions with chromatin remodelers.

## m ^6^A RNA modification in oligodendrocyte progression and homeostasis

*N* ^6^-methyladenosine (m ^6^A) is the most abundant internal modification of mRNA in eukaryotes. A methyl group can be added to the N ^6^ position of adenosines in specific sequences by m ^6^A methyltransferases (m ^6^A writers) such as METTL3 and METTL14 or removed by demethylases (m ^6^A erasers) such as FTO and ALKBH5. The effects of m ^6^A methylation on translation and RNA stability is mediated by m ^6^A-specific binding proteins (m ^6^A readers) including YTH-domain containing family proteins, hnRNP proteins, PRRC2A, and IGF2BP ^159– 161^.

Recent studies have revealed a differential m ^6^A methylation of core oligodendrocyte lineage genes during OPC differentiation, suggesting an important role for this process in OL differentiation ^76^. Deleting the METTL14 led to defects in OPC differentiation and hypomyelination at least in part by regulating alternative mRNA splicing in OL-expressing genes, including the paranodal protein NF155, which is critical for the establishment and maintenance of nodes of Ranvier ^76^. In addition, the m ^6^A RNA binding protein PRRC2A is highly expressed in OPCs during development in the white matter tracks. Both knockout and knockdown of PRRC2A in NPCs via *Nestin-Cre* or *Olig2-Cre* ^+^ oligodendrocyte lineage cells led to hypomyelination due to defects in OPC proliferation and differentiation ^77^. PRRC2A was shown to bind to the *Olig2* mRNA and further stabilize the expression of *Olig2* transcript in an m ^6^A-dependent manner. Knocking out the RNA demethylase FTO mimicked the effects of PRRC2A overexpression ^77^ and led to increased *Olig2* expression levels. These studies suggest a critical role for m ^6^A modification in the OL myelination process. The function of other mRNA modification enzymes remains to be determined in myelination and remyelination in the CNS.

## Conclusion and perspectives

Chromatin modifications and epigenetic regulation are crucial for oligodendrocyte fate specification, OPC proliferation, and oligodendrocyte differentiation ( Figure 1). Many members of chromatin modifiers discussed above have not yet been examined in the context of oligodendrocyte development and regeneration. In particular, although the major mediators of these developmental processes are being identified, the environmental influences that modulate the epigenetic mechanisms are very poorly understood. A better understanding of the mechanisms underlying the windows of epigenetic engagement will facilitate oligodendrocyte regeneration and remyelination.

Targeting epigenetic factors to influence OPC differentiation as a means to promote myelin regeneration after nerve injury or in demyelinating diseases is an exciting potential therapeutic avenue. In MS, for example, many demyelinating plaques still contain OPCs; however, these cells fail to differentiate to replace those lost. Additionally, oligodendrocyte loss and the subsequent loss of myelin sheaths have been implicated in Alzheimer’s disease ^55, 92, 162, 163^. Stimulating OPCs to proliferate and differentiate would be an exciting treatment option in slowing the disease progression.

In the future, it may prove fruitful to further scrutinize *in vivo* models of demyelinating diseases for temporal changes in chromatin landscape, structure, occupancy, and activity in response to myelin-promoting stimuli or pharmacological treatments, such as those used in MS disease-modifying therapies. Future work of exploring these various family members of chromatin modifiers and identifying specific epigenetic modifiers responsible for CNS myelination and remyelination will facilitate the development of effective treatments for developmental disorders and neurodegenerative diseases.
